# Chronic bronchiectasis complicated with benign jejuno‐ileal pneumatosis intestinalis

**DOI:** 10.1002/rcr2.985

**Published:** 2022-06-07

**Authors:** Sheng‐Huan Wei, Li‐Ting Huang, Tang‐Hsiu Huang

**Affiliations:** ^1^ Division of Chest Medicine, Department of Internal Medicine National Cheng Kung University Hospital, College of Medicine, National Cheng Kung University Tainan Taiwan; ^2^ Department of Diagnostic Radiology National Cheng Kung University Hospital, College of Medicine, National Cheng Kung University Tainan Taiwan; ^3^ Institute of Clinical Medicine College of Medicine, National Cheng Kung University Tainan Taiwan

**Keywords:** antibiotic‐associated diarrhoea, bronchiectasis, pneumatosis intestinalis

## Abstract

Patients with chronic bronchiectasis are susceptible to various respiratory complications. In this report, however, we describe a 53‐year‐old male with chronic bronchiectasis who developed extensive but asymptomatic jejuno‐ileal pneumatosis intestinalis. The patient did not have preceding pneumothorax or pneumomediastinum, and he did not receive cytotoxic or immunosuppressive therapy. Nor did he exhibit any clinical or radiographic evidence of intestinal ischaemia, obstruction or infection. Mucosal defects, due to his severe diarrhoea relating to the prolonged anti‐pseudomonal antibiotic treatment for his lungs, and the intestinal luminal pressure fluctuation, resulting from his exacerbated cough and from his frequent abdominal straining during defecation, were considered to have precipitated the condition. Following conservative treatment, the patient recovered well. In addition to adverse respiratory events, clinicians managing patients with bronchiectasis should also be alert to such an unusual extrapulmonary complication, because either neglecting the condition or unnecessary exploratory surgery may lead to hazardous outcomes.

## Graphical abstract

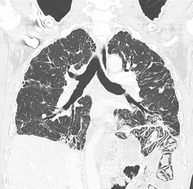

## INTRODUCTION

Patients with chronic bronchiectasis may experience exacerbations and develop various infectious or non‐infectious respiratory complications.^1^ Due to the enhanced risk of superimposed infection (especially by *Pseudomonas aeruginosa*), these patients are also likely to receive systemic antibiotic treatment (either prophylactic or eradicative) for an extended duration.^1^ In this report, we describe a case of chronic bronchiectasis with repeated episodes of pseudomonal pneumonia that was later complicated with extensive but asymptomatic pneumatosis intestinalis involving the small bowel. Without evidence of mesenteric ischaemia or infection, the exacerbated cough during pneumonia and the severe antibiotic‐associated diarrhoea were considered to have prompted the entry of luminal gas into the intestinal wall in this patient.^2^

## CASE REPORT

**FIGURE 1:**
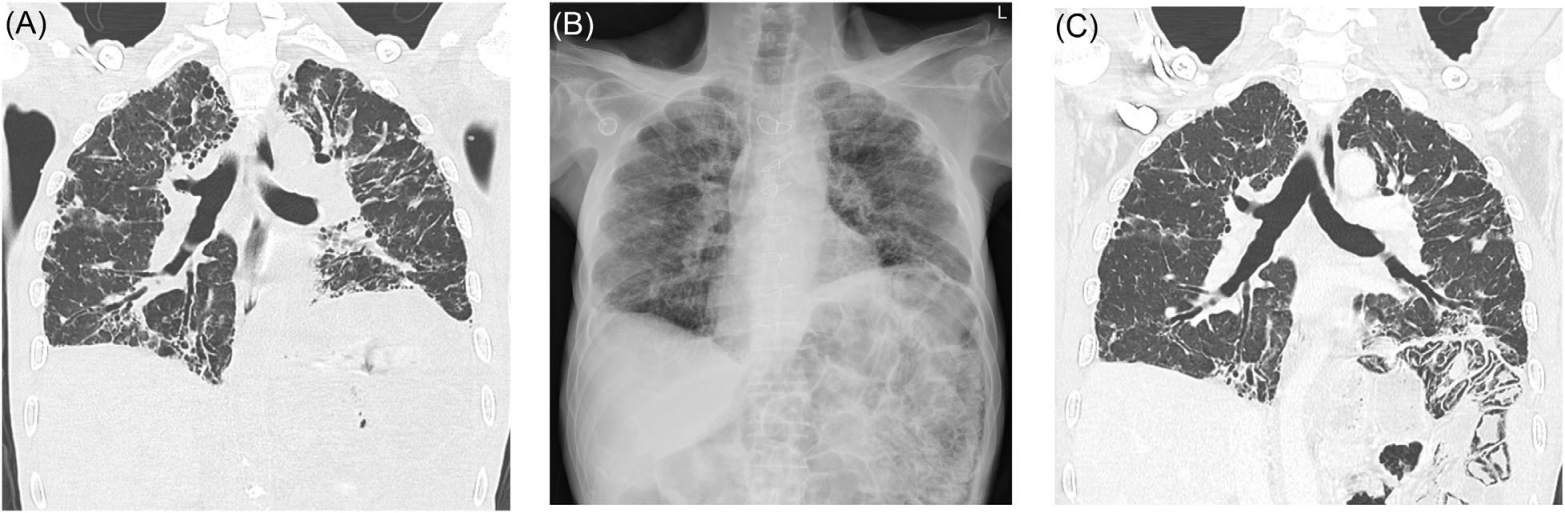
(A) A representative coronal‐view thoracic computed tomography (CT) image of the patient showing bilateral multi‐lobar bronchiectasis. (B) The follow‐up chest radiograph and (C) coronal‐view CT image incidentally revealed pneumatosis intestinalis, but not any evidence of pneumothorax or pneumomediastinum.

A 53‐year‐old male was admitted in August 2021 for incidentally found pneumatosis intestinalis. The patient had uneventful perinatal and growth histories. He had histories of invasive thymoma (for which he had received surgical resection and adjuvant chemotherapy 2 years earlier) and chronic bronchiectasis (Figure 1A) that had resulted from repeated episodes of lower airway infection complicating his chemotherapies. He was not receiving any immunosuppressant and exhibited no evidence of connective tissue disease or hypogammaglobulinaemia (as in Good's syndrome; Table 1).

**TABLE 1:** Results of pertinent blood and faecal analyses and microbiological studies

Blood biochemistry and serology	Results	Reference range
ANA	1:40 (−)	<1:80
Anti‐double‐stranded DNA (IU/ml)	1.2	<10
Anti‐Smith (SmDP) antibody (U/ml)	1.5	<7
Antinuclear RNP antibody (U/ml)	1.5	<5
Anti‐Ro/SSA antibodies (U/ml)	1.1	<7
Anti‐La/SSB antibodies (U/ml)	0.0	<7
MPO antibody (IU/ml)	0.2	<3.5
PR3 antibody (IU/ml)	0.2	<3.5
Scl‐70 antibody (U/ml)	1.0	<7
Anti‐histidyl‐tRNA synthetase antibody (U/ml)	0.0	<7
Immunoglobulin G (mg/dl)		751–1560
First check (January 2021)	2210	
Second check (October 2021)	2310	
Immunoglobulin A (mg/dl)		82–453
First check (January 2021)	163	
Second check (October 2021)	201	
Immunoglobulin M (mg/dl)		46–304
First check (January 2021)	167	
Creatinine (mg/dl)	0.48	0.70–1.20
Alanine transaminase (U/L)	14	≤50
Lipase (U/L)	23	13–60
C‐reactive protein (mg/L)	15.9	<8.0
Rheumatoid factor (U/L)	<20	<20
Lactate (mmol/L)	0.7	0.5–2.2
Venous blood gas		
pH	7.397	7.35–7.45
pCO_2_ (mmHg)	35.9	32–48
HCO_3_^−^ (mmHg)	27.2	21–29

Haemogram	Results	Reference range
WBC (/μl)	14,000	3400–9500
Neutrophils (%)	68.4	40.8–76.6
Lymphocytes (%)	23.3	15.4–47.0
Eosinophils (%)	0	0.4–7.5
Basophils (%)	0.1	0.2–1.7
Monocytes (%)	8.2	4.4–11.8
Haemoglobin (g/dl)	13.5	13.3–17.2
Platelets (/μl)	358,000	143,000–349,000

Faecal analysis	Results
Colour	Brown
Consistency	Semifluid
Red blood cells (/HPF)	Negative
Pus (/HPF)	Negative
Occult blood	Negative

Microbiological studies	Results
Blood bacterial culture	No growth
Faecal bacterial culture	No growth (including *Salmonella* and *Shigella* species)
*Clostridium difficile*	
GDH antigen	Negative
Toxins A and B antigens	Negative
Faecal parasites or ova	Negative

Abbreviations: ANA, antinuclear antibody; GDH, glutamate dehydrogenase; HPF, high‐power field; MPO, myeloperoxidase; pCO_2_, partial pressure of carbon dioxide; PR3, serine proteinase 3; RNP, ribonucleoprotein; WBC, white blood cell.

In the past year before this admission, he had been hospitalized four times due to superimposed lower airway *P*. *aeruginosa* infection and the associated acute exacerbation of bronchiectasis. Clinically, all four episodes of acute exacerbation manifested as severely aggravated productive cough and dyspnoea, which were treated by extended courses of antibiotics (including an initially eradicative intravenous anti‐pseudomonal agent that was subsequently followed by several weeks of consolidative oral ciprofloxacin plus inhalational amikacin or colistin). Shortly after the initiation of antibiotic treatment, the patient always developed diarrhoea, having five to seven times of loose‐to‐watery non‐bloody bowel movements per day. Meanwhile, he exhibited no evidence of arthritis, cholangitis, pancreatitis, erythema nodosum or ocular inflammation. His diarrhoea would quickly resolve following discontinuation of the antibiotics.

**FIGURE 2:**
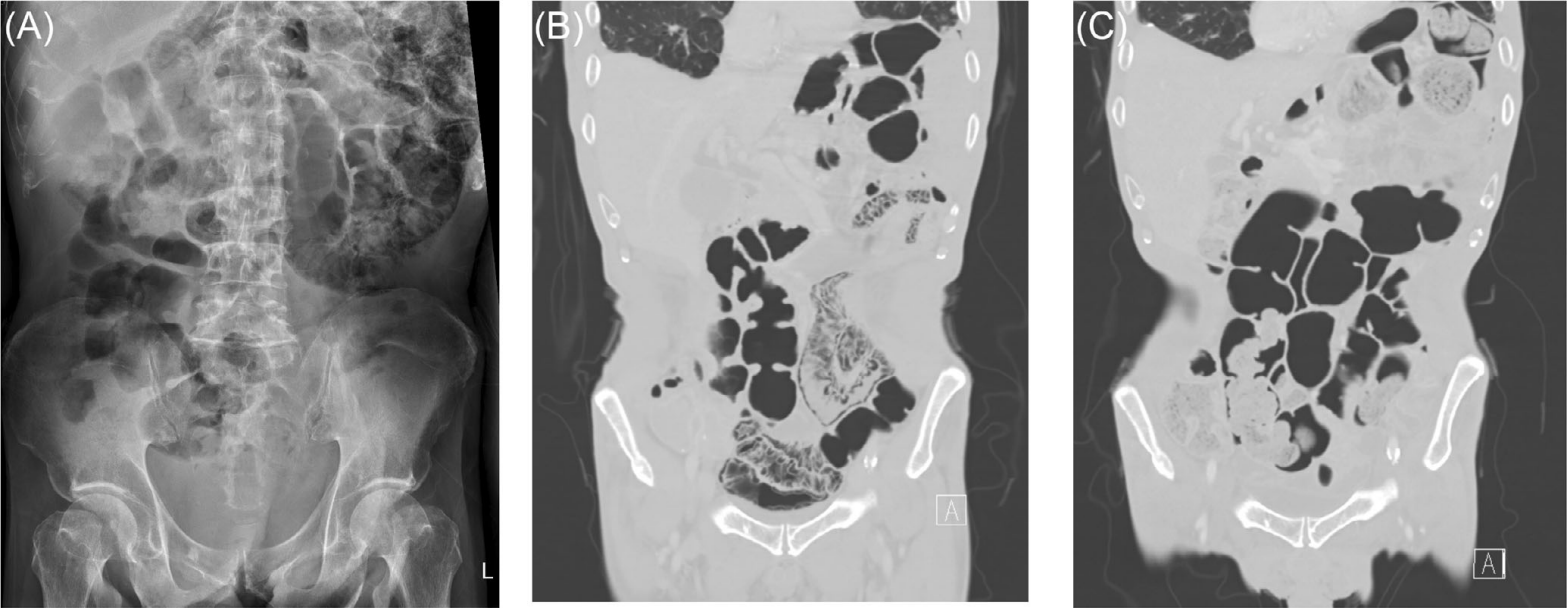
(A) The abdominal radiograph and (B) the coronal‐view abdominal computed tomography (CT) image showing the jejuno‐ileal pneumatosis intestinalis, which later completely resolved as revealed by (C) the follow‐up CT image 6 months later.

Three weeks before this admission, he had another episode of pseudomonal pneumonia, presenting with dyspnoea, worsening cough and increasing production of greenish sputum. Oxygenation, however, was well maintained using low‐flow nasal prongs. After the treatment, his pulmonary conditions improved, and the patient was discharged, continuing treatment with oral ciprofloxacin. Although he still had antibiotic‐associated diarrhoea, he did not complain about nausea, vomit, abdominal bloating or pain. He exhibited neither peritoneal sign nor haematochezia. Nor did he have abnormal symptoms in his eyes, joints and skin. The follow‐up chest radiograph and computed tomographic (CT) scan, however, revealed pneumatosis intestinalis (Figure 1B,C), which was clearly visualized on his abdominal radiograph and CT images, involving his jejunum and proximal ileum (Figure 2A,B). There was no pneumothorax, pneumomediastinum, pneumoperitoneum, pyloric stenosis, intestinal obstruction or mesenteric ischaemia. Blood tests revealed leucocytosis and elevated C‐reactive protein, but not acidaemia, lactatemia or renal injury. Repeated blood and faecal microbiological workups, including tests for *Clostridium difficile* toxins and microscopic examination for parasitic presence, showed no evidence of intestinal infection (Table 1). Following conservative treatment, including transient parenteral nutrition, supplementary oxygen and oral metronidazole, he recovered well. Another abdominal CT scan 6 months later showed complete resolution of pneumatosis intestinalis (Figure 2C).

## DISCUSSION

Ischaemia‐unrelated pneumatosis intestinalis may occur in patients with asthma, chronic obstructive pulmonary disease, cystic fibrosis and even tuberculosis.^2^, ^3^ There have been very few reports on pneumatosis intestinalis complicating non‐cystic fibrosis bronchiectasis. Doumit et al. reported a female patient with a history of chronic bronchiectasis who developed pneumatosis intestinalis that mainly involved her sigmoid colon and rectum, and that manifested as abdominal pain, bloating and diarrhoea. There was no evidence of bowel ischaemia, and the patient responded well to oral metronidazole. Clinical and radiographic details regarding her bronchiectasis, however, are not available from this report.^4^ Dawe and Akhtar described a male patient with diffuse pneumatosis intestinalis of the small bowel that progressed to pneumoperitoneum. Symptoms of the patient included anorexia, nausea, vomiting and diffuse and progressively worsening abdominal pain. The aetiology was later found to be mesenteric ischaemia, which was treated with angiographic balloon dilatation.^5^ The patient in our present report also had considerable involvement of the small bowel, but he exhibited neither clinical nor laboratory evidence of mesenteric ischaemia, parasitic infestation and infection (including *C*. *difficile*‐associated disease). Unlike in previously reported cases, his bronchiectasis was multi‐lobar and extensive, whereas his pneumatosis intestinalis was almost asymptomatic at the outset (except for the diarrhoea, but which had already preceded the pneumatosis by several months and was considered to be antibiotic‐associated) and underwent a rather benign course. We could not completely exclude the possibility of inflammatory bowel diseases (IBDs) because the patient refused colonoscopy. Nevertheless, the incidence and prevalence rates of IBD are very low in Taiwan.^6^ The clinical presentations of the patient were not compatible with that of IBD. The diarrhoea was temporally associated with antibiotic treatment and it resolved spontaneously after antibiotic discontinuation without any immunosuppressive therapy. There was no macro‐ or microscopic evidence of intestinal haemorrhage. Besides, the patient did not exhibit IBD‐associated extra‐intestinal manifestations, and he was serologically negative for anti‐neutrophil cytoplasmic antibodies (Table 1). He later received an oesophagogastroduodenoscopy in December 2021 (for peptic ulcers), wherein the gross appearance and biopsy histology of the gastric mucosa revealed no evidence of Crohn's disease. Therefore, IBD appears less likely in this patient.

Apart from ischaemia and infection, for patients with chronic respiratory diseases, one aetiologic theory proposes that barotrauma may allow the alveolar gas to leak through macro‐ or microscopic alveolo‐interstitial defects and eventually shift along the mediastinum down to the intestinal interstitium.^2^ This theory, however, cannot reasonably account for our case, considering the absence of any radiographic evidence of barotrauma, and also considering his disproportionally extensive pneumatosis intestinalis. In our opinion, the presence of gas in the intestinal walls of our patient might have resulted from a synergy of mucosal defects and intestinal luminal pressure fluctuation.^2^ Shortly before the diagnosis of pneumatosis intestinalis, the patient was just recovering from another episode of pneumonia, during which his cough aggravated. He was also suffering from frequent diarrhoea that was attributed to the ongoing anti‐pseudomonal antibiotic treatment. His exacerbated cough, and his frequent abdominal straining when defecation, might have caused pressure surges in the intestinal lumen, which would prompt luminal gas leakage through the mucosal defects resulting from the severe antibiotic‐associated diarrhoea.

Although our patient received non‐surgical managements and recovered well, surgical intervention may be needed in certain situations, and close monitoring is important. For patients with clinical or radiographic evidence of mechanical bowel disease (such as obstruction), iatrogenic bowel trauma (within the past 48 h), portal venous gas or strong suspicion of mesenteric ischaemia, exploratory surgery is indicated. Surgery is also considered for patients with pneumatosis intestinalis who respond poorly to conservative managements, and for those who have at least one of the following risk factors: age ≥ 60 years, significant leucocytosis (with a proposed cut‐off white blood cell count of >12,000/mm^3^), lactic acidosis, sepsis or peritonitis.^7^, ^8^

In conclusion, this case report shows that chronic bronchiectasis may be complicated with non‐ischaemic and non‐infectious pneumatosis intestinalis. Clinicians managing patients with bronchiectasis should be alert to such an unusual extrapulmonary complication, because either neglecting the condition or unnecessary exploratory surgery may lead to hazardous outcomes.

## CONFLICT OF INTEREST

None declared.

## ETHICS STATEMENT

The authors declare that appropriate written informed consent was obtained for the publication of this manuscript and accompanying images.

## Data Availability

Data are available on request due to privacy/ethical restrictions.
